# Clinical characteristics and distinct recovery trajectories of pain and disability after lumbar microdiscectomy in children and adolescents

**DOI:** 10.3389/fsurg.2026.1913776

**Published:** 2026-08-27

**Authors:** Muhammed Erkan Emrahoğlu, Mustafa Kavcar, Erdal Reşit Yılmaz, Hüseyin Bozkurt, Mehmet Kalan, Ahmet Günaydın, Hakan Yakupoğlu, Halil Gök, Levent Gürses, Emrah Keskin, Selim Gel, Mehmet Erhan Türkoğlu

**Affiliations:** 1 Department of Neurosurgery, Ankara Etlik City Hospital, Ankara, Türkiye; 2 Department of Orthopedics and Traumatology, Ankara Etlik City Hospital, Ankara, Türkiye; 3 Department of Neurosurgery, Zonguldak Bülent Ecevit University, Zonguldak, Türkiye; 4 Department of Neurosurgery, Trabzon University, Trabzon, Türkiye; 5 Department of Neurosurgery, Hacettepe University, Ankara, Türkiye

**Keywords:** adolescent, lumbar disc herniation, microdiscectomy, Oswestry Disability Index, pediatric neurosurgery

## Abstract

**Background:**

Pediatric and adolescent lumbar disc herniation (LDH) is uncommon, and recovery after microdiscectomy may differ across pain, disability, and outcome domains.

**Methods:**

This retrospective single-center case series included patients younger than 18 years of age who underwent microdiscectomy for LDH between 2022 and 2025 and had complete 12-month outcome data. Low back and radicular leg pain were assessed using the visual analog scale (VAS), disability using the Oswestry Disability Index (ODI), and global outcome using the modified MacNab criteria. Longitudinal changes were analyzed using Friedman tests and Holm-adjusted pairwise comparisons. A *post hoc* responder analysis applied adult-derived absolute thresholds and a reduction of at least 30%.

**Results:**

Seventeen patients were included (median age, 16 years; median follow-up, 21 months). The median low back pain VAS score increased from 4 preoperatively to 7 early postoperatively and then decreased to 1 at 3 and 12 months (overall *p* < 0.001). The median radicular leg pain VAS score decreased from 8 preoperatively to 2 early postoperatively and 1 at 3 and 12 months (overall *p* < 0.001). The median ODI score decreased from 50 preoperatively to 12 at 3 months and 6 at 12 months (overall *p* < 0.001), with further improvement between 3 and 12 months (*p* < 0.05). All evaluable patients met both responder criteria from baseline to 3 and 12 months. Modified MacNab categories did not change significantly (*p* = 0.313). No complications occurred; two patients developed recurrence and one underwent reoperation.

**Conclusions:**

Pain, disability, and global outcome followed distinct trajectories. These exploratory findings support separate assessment of recovery domains and require confirmation in larger multicenter cohorts.

## 1 Introduction

Lumbar disc herniation (LDH) is a common spinal disorder in adults and typically reflects age-related degenerative change (1, 2). In contrast, LDH is uncommon in children and adolescents and accounts for only 0.5%–6.8% of surgically treated cases (1, 3, 4). This epidemiological difference is related to the distinct anatomical and biomechanical properties of the growing spine (5, 6). In pediatric and adolescent cases, factors such as trauma, genetic predisposition, sports-related activity, biomechanical characteristics, and ring apophysis fracture may play a more prominent role than in adult degenerative disc disease (7, 8). In this age group, the relatively well-hydrated and less degenerative nature of the intervertebral disc may partly explain differences from adults in both disease behavior and response to treatment (1, 8).

In pediatric and adolescent patients without neurological deficit, conservative treatment is generally considered the first-line approach (1). However, in the presence of pain refractory to conservative treatment, substantial functional impairment, or neurological deficit, microdiscectomy has been reported to be a safe and effective surgical option with low complication rates (9, 10). Available studies have generally assessed surgical outcomes using pain, disability, complication, recurrence, and global clinical outcome measures (10, 11). In this study, we aimed to evaluate different dimensions of postoperative recovery in children and adolescents who underwent microdiscectomy for LDH by examining longitudinal changes in low back pain, radicular leg pain, the Oswestry Disability Index (ODI), and modified MacNab outcomes.

## 2 Materials and methods

### 2.1 Study design and patients

This was a single-center retrospective observational case series of children and adolescents who underwent microdiscectomy for LDH between October 2022 and March 2025. Patients were included if they were younger than 18 years at surgery and had complete data for the 12-month outcomes assessed in this study. Patients managed non-operatively, those with insufficient documentation of the index surgery, and those without reliable postoperative outcome verification were excluded. The study was approved by the local ethics committee, and the requirement for informed consent was waived because of the retrospective design.

### 2.2 Data collection and clinical–radiological assessment

Data were collected retrospectively from the hospital information system, discharge summaries, operative reports, outpatient records, and radiological images. Preoperative clinical variables and postoperative visual analog scale (VAS) scores were extracted from contemporaneous institutional records. ODI scores at 3 and 12 months were retrospectively calculated, and modified MacNab outcomes were classified, using the clinical information documented in the corresponding follow-up records. Because family history of lumbar disc herniation and recreational or professional sports participation might not have been systematically documented or queried at the initial assessment, these variables were additionally verified through telephone interviews conducted with the patient and one parent. Recurrence and reoperation status were also verified during these interviews because events occurring at another institution might not have been captured in our hospital records. All variables were entered into a predefined data collection form before analysis. Demographic and clinical variables included age, sex, height, body weight, body mass index, daily activity profile, trauma history, family history, presenting complaint, symptom duration, low back pain, radicular leg pain, sensory symptoms, motor deficit, reflex change, incontinence, and the straight leg raise test (SLR). Body mass index status was classified using age- and sex-specific BMI-for-age percentiles as underweight (<5th percentile), healthy weight (5th to <85th percentile), overweight (85th to <95th percentile), or obesity (≥95th percentile). Daily activity profile was classified as sedentary student, recreational athlete, professional athlete, or heavy worker/apprentice; professional athletes were licensed school or club athletes, whereas recreational athletes participated in regular non-professional sports or exercise. Trauma history was recorded when a high-energy trauma or fall onto the back preceded symptom onset. Family history of LDH was recorded separately for first- and second-degree relatives. The primary presenting complaint was categorized as one of the following: low back pain, radicular leg pain, combined presentation, motor deficit, or incontinence. Combined presentation was defined as the presence of both low back pain and radicular leg pain at presentation. Symptom duration was calculated in months from symptom onset to surgery. The operated disc level, side of herniation, and herniation morphology were recorded. Disc morphology was classified as bulging, protrusion, extrusion, or sequestration. Ring apophysis fracture and lumbosacral anatomical variants were recorded when identified on radiological imaging; available computed tomography scans were also reviewed for osseous findings.

### 2.3 Surgical procedure and follow-up

All procedures were performed by 8 different neurosurgeons. All patients underwent microsurgical lumbar discectomy at the index level under general anesthesia. After fluoroscopic confirmation of the surgical level, a unilateral posterior approach was used, and the herniated disc material compressing the dural sac or the affected nerve root was removed under microscopic visualization. No annular closure procedure or other pediatric-specific intraoperative modification intended to reduce recurrence was used. After removal of the compressive disc fragment, additional discectomy from the disc space was performed at the discretion of the operating surgeon. Postoperatively, all patients were advised to remain at home with restricted activity for 1 month, were provided 2 months of leave from school or work, and were advised to defer return to sports for 3 months. Surgical indication was classified as refractory pain, neurological deficit, or functional impairment. Intraoperative and postoperative complications, length of hospital stay, follow-up duration, recurrence, reoperation, and mortality were recorded. Recurrence was defined as symptomatic recurrent disc herniation at the same level during follow-up, confirmed radiologically. Reoperation and the indication for reoperation were recorded separately.

### 2.4 Outcome measures

Pain severity was assessed using the VAS, scored from 0 to 10. Low back pain and radicular leg pain were recorded separately. VAS scores were obtained preoperatively, during the early postoperative bedside assessment, and at postoperative 3 and 12 months. Functional status was assessed using the Oswestry Disability Index; scores were expressed as percentages and recorded preoperatively and at 3 and 12 months postoperatively. ODI categories were defined as minimal disability (0–20), moderate disability (21–40), severe disability (41–60), crippled (61–80), and bed-bound (81–100). Global clinical outcome was assessed at 3 and 12 months using the modified MacNab criteria and classified as excellent, good, fair, or poor. Excellent and good outcomes indicated complete or marked symptomatic improvement with return to daily activities, whereas fair and poor outcomes indicated persistent functional impairment, insufficient improvement, or clinical deterioration.

The primary outcome measures were changes over time in low back pain VAS, radicular leg pain VAS, and ODI scores. Secondary outcome measures were modified MacNab outcome, recovery of preoperative motor deficits, recurrence, reoperation, perioperative complications, length of hospital stay, and mortality.

### 2.5 Patient-level clinical relevance analysis

To complement the group-level longitudinal analyses, we performed a *post hoc* patient-level responder analysis for low back pain VAS, radicular leg pain VAS, and ODI scores. Absolute improvement was calculated by subtracting the follow-up score from the corresponding reference score. For changes from the preoperative assessment to 3 and 12 months, the minimum clinically important difference (MCID) was defined as a reduction of at least 1.2 points for the low back pain VAS score, 1.6 points for the radicular leg pain VAS score, and 12.8 points for the ODI score, as described by Copay et al. in adult patients who had undergone lumbar spine surgery (12). A second responder criterion was defined as a reduction of at least 30% from the reference score, based on the approach described by Asher et al. in adult patients who had undergone lumbar spine surgery (13). Relative reduction was calculated as [(reference score−follow-up score)/reference score] × 100. The preoperative score served as the reference for the preoperative-to-3-month and preoperative-to-12-month analyses. To assess additional recovery after the early postoperative period, changes from 3 to 12 months were evaluated using the 3-month score as the reference. Because the absolute MCID thresholds and the 30% relative reduction criterion were developed in adult populations primarily for comparisons between the preoperative baseline and the postoperative follow-up, their application to the 3-to-12-month interval was considered an exploratory interval responder analysis rather than a formally validated MCID analysis. Outcomes with a reference score of 0 were not evaluable for reduction and were excluded from the denominator for the corresponding outcome and interval; these observations were not classified as non-responders.

### 2.6 Statistical analysis

Continuous variables were presented as median and interquartile range (IQR), with overall range when appropriate, and categorical variables as number and percentage. Overall time effects for low back pain VAS, radicular leg pain VAS, and ODI scores were assessed using the Friedman test. When significant, pairwise comparisons were performed using the exact Wilcoxon signed-rank test with Holm correction for multiple testing. All *p*-values reported for pairwise *post hoc* comparisons in the text and tables are Holm-adjusted *p*-values. Modified MacNab categories were analyzed as an ordinal variable, and changes between postoperative 3 and 12 months were assessed using the exact Wilcoxon signed-rank test. Effect size was reported as Kendall's *W* for omnibus repeated-measures analyses and as the rank-biserial correlation coefficient for pairwise comparisons. Exploratory associations between preoperative symptom duration and the 12-month low back pain VAS, radicular leg pain VAS, and ODI scores and modified MacNab outcomes were assessed using Spearman rank correlation. Patient-level responder outcomes were summarized as the number of responders divided by the number of patients evaluable for the corresponding outcome and interval, with results presented as *n*/*N* (%). Responder proportions were analyzed descriptively; no hypothesis testing was applied because of the small sample size and the *post hoc*, exploratory nature of these analyses. A two-sided *p*-value of less than 0.05 was considered statistically significant.

### 2.7 Ethical approval

All procedures performed in this study involving human participants received ethical approval from the Scientific Research Evaluation and Ethics Committee of Ankara Etlik City Hospital, approval number AEŞH-BADEK1-2026-368, and were conducted in accordance with the 1964 Helsinki Declaration and its later amendments.

## 3 Results

### 3.1 Cohort characteristics and perioperative outcomes

**Figure 1:**
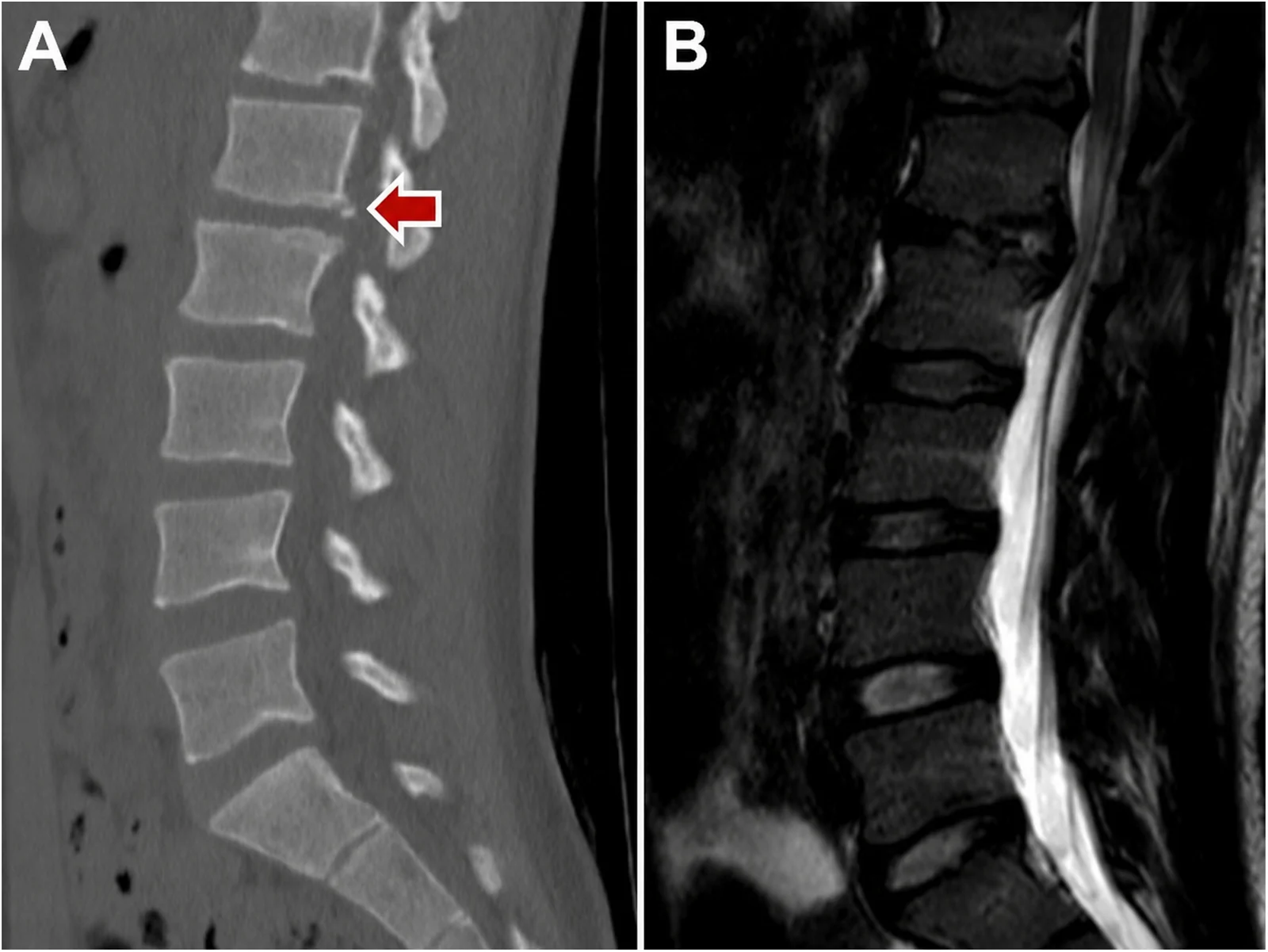
Ring apophysis fracture associated with pediatric lumbar disc herniation at L1–2. This case was selected because it clearly demonstrates the ring apophysis fracture; however, upper lumbar disc herniations were exceptional in our cohort. **(A)** A sagittal computed tomography image demonstrates a small posterior osseous fragment at the L1–2 level, indicated by the red arrow, consistent with a ring apophysis fracture. **(B)** A sagittal T2-weighted magnetic resonance image of the same patient demonstrates an associated posteriorly extruded disc herniation at L1–2 with indentation of the ventral thecal sac. This figure illustrates the complementary role of computed tomography in depicting the ring apophysis fracture and magnetic resonance imaging in demonstrating the associated disc herniation and neural compression.

A total of 17 pediatric and adolescent patients were included. Median follow-up was 21 months (IQR 17–26; range 13–32). Median age was 16 years (IQR 15–17; range 11–17), and nine patients (52.9%) were male. Median BMI was 25.9 kg/m^2^ (IQR 21.6–29.3; range 18.4–44.1). The activity profile consisted of nine sedentary students (52.9%), two recreational athletes (11.8%), five professional athletes (29.4%), and one heavy worker or apprentice (5.9%). Median symptom duration was 6 months (IQR 1–12; range 1–36). The most common primary presenting complaint was combined presentation (7/17, 41.2%), followed by radicular leg pain (6/17, 35.3%). Low back pain was present in 12 patients (70.6%), radicular pain in 14 (82.4%), sensory symptoms in four (23.5%), a positive SLR test result in 11 (64.7%), and motor deficit in five (29.4%). A family history in a first-degree relative was present in one patient (5.9%) and in a second-degree relative in five (29.4%). No history of trauma, sensory deficit, reflex change, or incontinence was identified. L4–5 and L5-S1 were the most frequently affected levels, each in seven patients (41.2%); the remaining three cases involved L1–2, L2–3, and L3–4. The herniation side was right in nine patients (52.9%) and left in eight (47.1%). Morphologically, seven cases (41.2%) were classified as extrusion, five (29.4%) as protrusion, three (17.6%) as sequestration, and two (11.8%) as bulging. Ring apophysis fracture was present in two patients (11.8%) (Figure 1).

The most common surgical indication was functional impairment (8/17, 47.1%), followed by refractory pain (5/17, 29.4%) and neurological deficit (4/17, 23.5%). Additional discectomy from the disc space was performed in nine patients (52.9%) and was not performed in eight patients (47.1%). Median hospital stay was 2 days (IQR 1–2; range 1–6). No intraoperative or postoperative complications were observed. All five patients with preoperative motor deficits had incomplete weakness, and none presented with foot drop. Complete motor recovery was documented before discharge in three patients and by the 3-month follow-up in the remaining two patients. No patient had a persistent motor deficit at 3 months. During follow-up, recurrence developed in two patients (11.8%), both of whom had undergone additional disc-space discectomy, and one patient (5.9%) underwent reoperation. No comparative analysis was performed because of the small number of recurrence events. No mortality event occurred.

**Figure 2:**
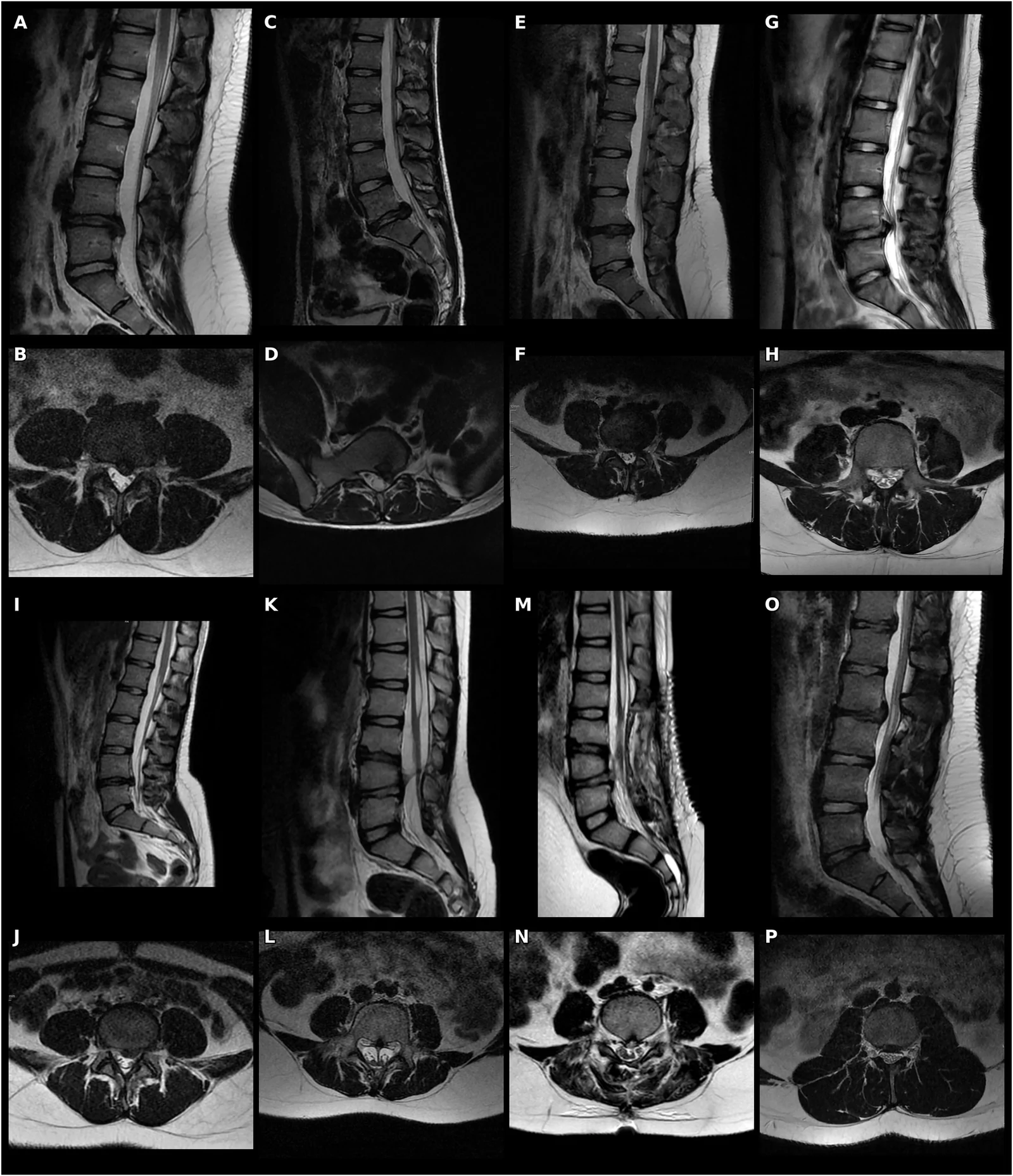
Representative sagittal and axial T2-weighted magnetic resonance images illustrating the clinical and radiological heterogeneity of pediatric and adolescent lumbar disc herniation. **(A,B)** A 14-year-old boy presented with low back pain, left radiculopathy, left-leg numbness, and dorsiflexion weakness. The images demonstrate an extruded L4–5 disc herniation compressing the left L5 nerve root. **(C,D)** A 16-year-old boy presented with acute left radicular pain and numbness without motor deficit. The images demonstrate an extruded left L5–S1 disc herniation with a marked compression of the left S1 nerve root. **(E,F)** A 14-year-old girl who was a competitive volleyball player presented with low back pain and numbness extending along the lateral aspect of the left leg to the dorsum of the foot and toes, without motor deficit. The images demonstrate a left-predominant central L4–5 disc protrusion compressing the thecal sac and left L5 nerve root. **(G,H)** A 13-year-old boy, the youngest patient in our cohort with no history of prior spinal surgery, presented with severe low back pain, right radicular pain, poorly localized numbness in both legs, and weakness of right knee extension and ankle dorsiflexion. The images demonstrate a cranially migrated central L4–5 disc extrusion causing central canal narrowing and bilateral neural compression. **(I,J)** A 14-year-old girl with no regular sports participation presented with low back and radicular pain without motor deficit. She was the only patient in the cohort with a first-degree family history of adolescent lumbar disc herniation; her mother had undergone surgery for lumbar disc herniation at 16 years of age and subsequently required additional revision and stabilization procedures. The images demonstrate an extruded left L4–5 disc herniation compressing the left L5 nerve root. **(K–N)** An 11-year-old girl had previously undergone surgery for tethered cord and type I split cord malformation. Prerepair images **(K,L)** demonstrate a low-lying cord and split cord malformation at L4–5. Six months after repair, she presented with severe left radiculopathy, numbness over the dorsum of the foot and dorsolateral leg, and dorsiflexion weakness. The subsequent images **(M,N)** demonstrate the repaired malformation, with both hemicords contained within a single dural sac, and a sequestered L4–5 disc fragment compressing the left L5 nerve root. Microdiscectomy was performed without stabilization, and she remained asymptomatic at 2-year follow-up. **(O,P)** A 15-year-old boy who was a competitive kickboxer presented with severe low back pain, bilateral anterior-thigh paresthesia, and inability to perform squats despite no definite motor deficit on routine examination. Imaging demonstrates disc protrusions at T12–L1 and L2–3, with the symptomatic lesion at L2–3. After right L2–3 microdiscectomy, he returned to competitive sports and remained asymptomatic at the 18-month follow-up.

Baseline demographic, clinical, radiological, and perioperative characteristics are summarized in Table 1. Representative magnetic resonance imaging findings illustrating the demographic, clinical, and radiological heterogeneity of the selected patients are presented in Figure 2.

**Table 1:** Baseline clinical, radiological, and perioperative characteristics.

Variable	Value
Demographics	
Age, years	16 [15–17]; 11–17
Male sex	9 (52.9%)
Body mass index (kg/m^2^)	25.9 [21.6–29.3]; 18.4–44.1 Underweight: 2 (11.8%); healthy weight: 9 (52.9%); overweight: 4 (23.5%); obesity: 2 (11.8%)
Activity profile	Sedentary student: 9 (52.9%); recreational athlete: 2 (11.8%); professional athlete: 5 (29.4%); heavy worker/apprentice: 1 (5.9%)
Clinical presentation	
Symptom duration (months)	6 [1–12]; 1–36
Primary presenting complaint	Combined: 7 (41.2%); radicular leg pain: 6 (35.3%); low back pain: 3 (17.6%); motor deficit: 1 (5.9%)
Low back pain	12 (70.6%)
Radicular pain	14 (82.4%)
Sensory symptoms	4 (23.5%)
Positive straight leg raise test	11 (64.7%)
Motor deficit	5 (29.4%)
Family history of lumbar disc herniation	First-degree: 1 (5.9%); second-degree: 5 (29.4%)
Radiological and perioperative findings	
Disc level	L4–5: 7 (41.2%); L5-S1: 7 (41.2%); L1–2: 1 (5.9%); L2–3: 1 (5.9%); L3–4: 1 (5.9%)
Herniation side	Right: 9 (52.9%); left: 8 (47.1%)
Herniation type	Extrusion: 7 (41.2%); protrusion: 5 (29.4%); sequestration: 3 (17.6%); bulging: 2 (11.8%)
Ring apophyseal fracture	2 (11.8%)
Surgical indication	Functional impairment: 8 (47.1%); refractory pain: 5 (29.4%); neurological deficit: 4 (23.5%)
Additional disc-space discectomy	Performed: 9 (52.9%); not performed: 8 (47.1%)
Length of hospital stay, days	2 [1–2]; 1–6
Follow-up, months	21 [17–26]; 13–32
Safety outcomes	
Intraoperative complication	0 (0%)
Postoperative complication	0 (0%)
Recurrence	2 (11.8%)
Reoperation	1 (5.9%)
Mortality	0 (0%)

Continuous variables are presented as median [IQR]; range. Categorical variables are presented as *n* (%).

### 3.2 Longitudinal clinical outcomes

Longitudinal recovery patterns for pain, disability, and global clinical outcome are presented in Table 2, and pairwise *post hoc* comparisons are presented in Table 3. Low back pain VAS scores changed significantly over time (Friedman test, *p* < 0.001; Kendall's *W* = 0.602). The median low back pain VAS score increased from 4 preoperatively to 7 in the early postoperative period (*p* < 0.05), before decreasing to 1 at both 3 and 12 months. Compared with the preoperative period, significant decreases were observed at 3 months (median 1; *p* < 0.05) and 12 months (median 1; *p* < 0.05). The difference between 3 and 12 months was not significant (adjusted *p* = 0.219). All 12 patients with preoperative low back pain had lower VAS scores at both 3 and 12 months than at baseline.

**Table 2:** Descriptive longitudinal outcome measures.

Outcome measure	Preop.	Early postop.	3 months	12 months	Overall *p*-value	Effect size
Back pain VAS score	4 [0–7]	7 [6–8]	1 [1–2]	1 [1–1]	<0.001*	Kendall's *W* = 0.602
Radicular leg pain VAS score	8 [8–9]	2 [1–2]	1 [1–2]	1 [1–1]	<0.001*	Kendall's *W* = 0.474
ODI score	50 [44–58]	—	12 [10–14]	6 [5–8]	<0.001*	Kendall's *W* = 0.945
MacNab outcome	—	—	Excellent: 7 (41.2%); Good: 10 (58.8%)	Excellent: 11 (64.7%); Good: 5 (29.4%); Fair: 1 (5.9%)	0.313^a^	*r* = −0.600

*p < 0.05 was considered statistically significant.

VAS, visual analog scale; ODI, Oswestry Disability Index; Preop., preoperative; Postop., postoperative; MacNab, modified MacNab criteria. Values are presented as median [IQR] for continuous variables and *n* (%) for categorical variables. For the back pain VAS, radicular leg pain VAS, and ODI scores, the reported overall *p*-values represent the overall time effect across all available assessment points. These *p*-values indicate whether a significant change occurred at any time point but do not specify between which time points the differences occurred. Pairwise *post hoc* comparisons were therefore performed separately and are presented in Table 3. Overall time effects for the VAS and ODI scores were evaluated using the Friedman test.

a For modified MacNab criteria, the reported *p*-value reflects the paired comparison between the 3- and 12-month distributions using ordinal paired analysis; it is not an omnibus time-effect test.

**Table 3:** Pairwise *post hoc* comparisons of longitudinal outcome measures.

Outcome measure	Comparison	Median change [IQR]	Adjusted *p*-value	Effect size (*r*)
Back pain VAS score				
	Preop. vs. early postop.	2 [1–6]	<0.05*	0.817
	Preop. vs. 3 months	−3 [−5 to −1]	<0.05*	−0.804
	Preop. vs. 12 months	−3 [−5 to −1]	<0.05*	−0.804
	3 vs. 12 months	0 [−1 to 0]	0.219	−0.571
Radicular leg pain VAS score				
	Preop. vs. early postop.	−7 [−7 to −6]	<0.001*	−0.922
	Preop. vs. 3 months	−7 [−7 to −6]	<0.001*	−0.983
	Preop. vs. 12 months	−7 [−8 to −6]	<0.001*	−0.983
	3 vs. 12 months	0 [−1 to 0]	0.129	−0.600
ODI score				
	Preop. vs. 3 months	−36 [−44 to −34]	<0.001*	−1.000
	Preop. vs. 12 months	−42 [−50 to −37]	<0.001*	−1.000
	3 vs. 12 months	−6 [−6 to −4]	<0.05*	−0.778
MacNab outcome				
	3 vs. 12 months	—	0.313	−0.600

*p < 0.05 was considered statistically significant.

VAS, visual analog scale; ODI, Oswestry Disability Index; Preop., preoperative; Postop., postoperative; MacNab, modified MacNab criteria. Pairwise comparisons were performed using the exact Wilcoxon signed-rank test. The reported *p*-values for the back pain VAS, radicular leg pain VAS, and ODI scores were adjusted for multiple comparisons within each outcome family using the Holm method. The MacNab comparison reflects paired ordinal analysis between the 3- and 12-month assessments. The effect size reflects the magnitude and direction of change and not statistical power. Median change was calculated as the later time point minus the earlier time point.

Radicular leg pain VAS scores also showed a significant overall time effect (Friedman test, *p* < 0.001; Kendall's *W* = 0.474). The median radicular leg pain VAS score decreased from 8 preoperatively to 2 in the early postoperative period (*p* < 0.001) and remained low at 3 and 12 months, with a median score of 1 at both time points. Compared with baseline, this reduction persisted at 3 months (median 1; *p* < 0.001) and 12 months (median 1; *p* < 0.001). No additional significant change was observed between 3 and 12 months (*p* = 0.129). All 14 patients with preoperative radicular pain showed improvement from baseline in the early postoperative period and at both follow-up visits.

The ODI scores changed significantly over time (Friedman test, *p* < 0.001; Kendall's *W* = 0.945). The median ODI score decreased from 50 preoperatively to 12 at 3 months (*p* < 0.001) and to 6 at 12 months (*p* < 0.001). The ODI score continued to improve between 3 and 12 months, with an additional significant decrease during this interval (*p* < 0.05). Preoperatively, ODI categories were severe in 14 patients (82.4%), moderate in 2 (11.8%), and crippled in 1 (5.9%); at 3 months, all patients were in the minimal disability category. At 12 months, 16 patients (94.1%) were in the minimal disability category and one (5.9%) was in the moderate disability category.

According to the modified MacNab criteria, outcomes at 3 months were excellent in seven patients (41.2%) and good in 10 (58.8%). At 12 months, modified MacNab outcomes were excellent in 11 patients (64.7%), good in five (29.4%), and fair in one (5.9%). In contrast to the ODI scores, modified MacNab categories did not change significantly between 3 and 12 months (*p* = 0.313).

Preoperative symptom duration was not significantly associated with the 12-month low back pain VAS score (*ρ *= 0.28, *p* = 0.274), the radicular leg pain VAS score (*ρ *= −0.01, *p* = 0.966), the ODI score (*ρ *= −0.07, *p* = 0.793), or the modified MacNab outcome (*ρ *= 0.07, *p* = 0.790).

### 3.3 Patient-level responder analysis

At both 3 and 12 months, all patients evaluable for improvement from the preoperative assessment met both the absolute and the relative responder criteria: 12 of 12 patients (100%) for the low back pain VAS score, 14 of 14 (100%) for the radicular leg pain VAS score, and 17 of 17 (100%) for the ODI score. Five patients with a preoperative low back pain score of 0 and 3 patients with a preoperative radicular leg pain score of 0 were not evaluable for reduction in the corresponding outcome and were excluded from those denominators.

In the exploratory analysis of additional change from 3 to 12 months, the adult-derived absolute point-change threshold was exceeded by two of 17 patients (11.8%) for low back pain, two of 15 (13.3%) for radicular leg pain, and one of 17 (5.9%) for the ODI. A reduction of at least 30% from the 3-month score occurred in six of 17 patients (35.3%) for low back pain, eight of 15 (53.3%) for radicular leg pain, and 16 of 17 (94.1%) for the ODI. Two patients with a 3-month radicular leg pain score of 0 were not evaluable for interval reduction. Patient-level responder results are presented in Table 4.

**Table 4:** Patient-level responder analysis for pain and disability outcomes.

Outcome	Interval	Evaluable patients, *N*	Absolute threshold achieved, *n*/*N* (%)	≥30% reduction achieved, *n*/*N* (%)
Low back pain VAS score	Preoperative to 3 months	12	12/12 (100)	12/12 (100)
Low back pain VAS score	Preoperative to 12 months	12	12/12 (100)	12/12 (100)
Low back pain VAS score	3–12 months	17	2/17 (11.8)	6/17 (35.3)
Radicular leg pain VAS score	Preoperative to 3 months	14	14/14 (100)	14/14 (100)
Radicular leg pain VAS score	Preoperative to 12 months	14	14/14 (100)	14/14 (100)
Radicular leg pain VAS score	3–12 months	15	2/15 (13.3)	8/15 (53.3)
ODI score	Preoperative to 3 months	17	17/17 (100)	17/17 (100)
ODI score	Preoperative to 12 months	17	17/17 (100)	17/17 (100)
ODI score	3–12 months	17	1/17 (5.9)	16/17 (94.1)

Absolute thresholds were defined as reductions of at least 1.2 points for the low back pain VAS score, 1.6 points for the radicular leg pain VAS score, and 12.8 points for the ODI score. Relative response was defined as a reduction of at least 30% from the reference score. The preoperative score was used as the reference for preoperative-to-3-month and preoperative-to-12-month analyses; the 3-month score was used for the exploratory 3-to-12-month analysis. Outcomes with a reference score of 0 were not evaluable and were excluded from the corresponding denominator. The 3-to-12-month analysis represents an exploratory interval assessment and not a validated minimum clinically important difference analysis.

## 4 Discussion

### 4.1 Principal findings

In this cohort, postoperative recovery after microdiscectomy for LDH showed different temporal patterns across pain, functional status, and global clinical outcome. Radicular leg pain decreased markedly in the early postoperative period, whereas low back pain transiently increased at the early postoperative assessment and then declined during follow-up. Functional improvement became evident by 3 months and continued through 12 months. In contrast, modified MacNab categories did not change significantly between 3 and 12 months. Within the limits of this small cohort, these observations suggest that postoperative recovery may be better characterized by assessing pain components and disability separately from broad global outcome categories. These findings should be considered exploratory rather than confirmatory.

### 4.2 Clinical and radiological context

The clinical and radiological profile of our cohort supports the view that pediatric LDH requiring surgical treatment occurs predominantly in late adolescence. Previous series have similarly reported that childhood LDH is uncommon, that surgically treated cases are concentrated mainly in the adolescent age group, and that L4–5 and L5–S1 are the predominant levels (9, 14–16). In our series, the presence of upper lumbar cases despite the predominance of lower lumbar levels indicates that levels outside the typical adult pattern should not be overlooked in children and adolescents. Kumar et al. and Gennuso et al. also emphasized that clinical suspicion in pediatric LDH should not be limited to the lower lumbar discs (2, 4).

The presenting features were consistent with those of adolescent patients with LDH referred for surgery. Combined low back and radicular leg pain and isolated radicular leg pain were the predominant presentations; a positive SLR test result was common, motor deficit was less frequent, and incontinence was not observed. Raghu et al. reported that low back pain, sciatica, and a positive SLR test are typical findings in surgically treated pediatric LDH, whereas autonomic findings are uncommon (8). Cahill et al. and Montejo et al. also showed in pediatric microdiscectomy series that radicular pain is one of the main clinical components of the surgical indication (9, 17). Kotil et al. reported that cauda equina presentation is possible but exceptional in children; the absence of sphincter dysfunction in our series supports this rarity (18). Preoperative symptom duration was not significantly associated with 12-month pain, disability, or modified MacNab outcomes in our cohort. Durham et al. reported no identifiable predictors of poor long-term outcome or reoperation in a pediatric surgical series (19). More recently, Høstmælingen et al. reported favorable 3-month recovery despite a median interval of 12 months from symptom onset to surgery, with a reduction in back pain in 95.2% of patients and a relief of radicular symptoms in all patients; however, these findings and ours should be interpreted cautiously because of the small cohort sizes (20).

The activity profile and family history findings indicate that a single risk model is insufficient to explain pediatric LDH. Although our series included professional and recreational athletes, a substantial proportion of patients were sedentary students, and no patient had a history of high-energy trauma. Shimony et al. evaluated the relationship between sports participation, BMI, and treatment success in adolescent disc disease, whereas Singhal et al. suggested that trauma and genetic predisposition may contribute to pediatric LDH (7, 11). Our findings suggest that mechanical loading may contribute in some patients, but the disease cannot be explained solely by athletic participation or overt trauma. BMI status was classified using age- and sex-specific percentiles and reported descriptively; however, the small sample size and absence of a control group precluded evaluation of BMI as a risk factor for LDH or as a predictor of postoperative outcomes. Although Qu et al. specifically examined endoscopic surgical outcomes in obese adolescents, our study was not designed to establish a causal relationship between BMI and outcome (21). Cases with ring apophysis fracture highlight the importance of the osseous component in pediatric LDH. Singhal et al. reported that ring apophysis fracture may be more common in children than in adults and that the diagnosis may be missed when CT is not performed (7). Because CT was not obtained systematically in all patients in our series, the true rate may have been underestimated. Therefore, although MRI remains the primary modality for evaluating neural compression, CT has complementary value when a posterior osseous fragment, a calcified component, or an atypical upper lumbar herniation is suspected (6).

### 4.3 Pain recovery and surgical approach

Pain outcomes were among the clearest findings of this study. Radicular leg pain resolved early and remained low, consistent with the relief of nerve root compression. Favorable pain and functional outcomes have also been reported after pediatric tubular and endoscopic discectomy. Montejo et al. reported excellent or good outcomes in 91% of patients after tubular minimally invasive microdiscectomy, an approach designed to preserve paraspinal muscle attachments and limit bone removal (17). Wang et al. found that both back and leg pain decreased from the first postoperative day and continued to decrease during follow-up after a percutaneous endoscopic interlaminar discectomy (22). Lin et al. likewise reported a marked reduction in back pain, leg pain, and disability after a percutaneous endoscopic lumbar discectomy (23).

Low back pain in our cohort followed a different early course, increasing during the immediate bedside postoperative assessment before declining by 3 months. Comparative adolescent studies provide a plausible explanation for this difference. Yu et al. reported lower early postoperative pain and better short-term functional outcomes after a percutaneous endoscopic discectomy than after open discectomy, although later outcomes were similar (3). Li et al. also reported less early low back pain after a percutaneous endoscopic discectomy than after microendoscopic discectomy and related this difference to reduced paraspinal muscle disruption with the smaller working corridor (24). The transient early increase observed in our cohort may therefore reflect both the timing of assessment and incisional or paraspinal pain associated with the conventional posterior microscopic approach. Because our study did not include a tubular or endoscopic comparison group, this interpretation remains provisional and should not be considered evidence of a technique-specific effect.

### 4.4 Functional recovery and clinical relevance

The patient-level responder analysis provided additional clinical context. Among patients with non-zero preoperative scores, all patients exceeded both the adult-derived absolute threshold and the 30% relative reduction criterion at 3 and 12 months for low back pain (12/12) and radicular leg pain (14/14). Thus, the statistically significant group-level changes were accompanied by a clinically substantial improvement in every evaluable patient. Further pain reduction after 3 months was less uniform. Between 3 and 12 months, two of 17 patients exceeded the absolute low back pain threshold and six of 17 had a reduction of at least 30%; the corresponding values for radicular leg pain were two of 15 and eight of 15. These findings suggest that most pain relief in our cohort occurred within the first 3 postoperative months, leaving limited room for additional absolute change thereafter.

The divergence between the ODI and the modified MacNab findings represents one of the main functional observations of this study. The ODI score continued to improve from 3 to 12 months, suggesting that daily functional recovery may extend beyond the period of early pain relief. Over the same interval, the modified MacNab distribution did not show a statistically detectable change. This finding should not be interpreted as evidence that global clinical status remained unchanged; rather, the broad ordinal categories may have been less sensitive than the continuous ODI scale to smaller late functional gains, particularly in this small cohort, in which most patients were already classified as having good or excellent outcomes at 3 months. Than et al. reported substantial heterogeneity in the outcomes used across pediatric and adolescent discectomy studies, including measures of pain, disability, complications, and global satisfaction (10). Cahill et al. and Montejo et al. also reported generally favorable clinical outcomes after pediatric microdiscectomy (9, 17). Our findings add that measurable reduction in disability may continue through 12 months even when broad global outcome categories show no statistically detectable interval change.

The patient-level responder analysis further clarified the clinical relevance of the ODI trajectory. All 17 patients exceeded both the adult-derived 12.8-point absolute threshold and the 30% relative reduction criterion from the preoperative assessment to 3 and 12 months. In the exploratory analysis of additional recovery between 3 and 12 months, only one of 17 patients exceeded a further absolute reduction of 12.8 points, whereas 16 of 17 achieved a reduction of at least 30% relative to their 3-month ODI score. The low rate of response according to the fixed absolute threshold does not contradict the statistically significant continued reduction in the ODI score. By 3 months, disability scores had already declined substantially; in 10 of 17 patients, the 3-month ODI score was below 12.8, making a further reduction of that magnitude mathematically impossible even if the 12-month score reached 0. The relative criterion was therefore more sensitive to smaller absolute changes that represented substantial improvement in relation to the disability remaining at 3 months. Because both criteria were developed in adult lumbar surgery populations and their application to change between 2 postoperative assessments has not been formally validated, the 3-to-12-month findings should be interpreted as an exploratory interval analysis rather than formal MCID attainment (12, 13). These findings also help explain why the continuous ODI measure identified late functional gains that were not reflected by a statistically detectable change in modified MacNab categories.

### 4.5 Clinical implications and safety

Taken together, the distinct trajectories observed for radicular leg pain, low back pain, and disability may help clinicians interpret the timing of recovery after pediatric lumbar microdiscectomy. In this cohort, radicular pain resolved early, low back pain could transiently increase immediately after surgery before subsiding, and functional improvement continued through 12 months even after pain scores had largely stabilized. These patterns may inform both preoperative counseling and postoperative follow-up by helping patients and parents distinguish expected early postoperative symptoms from outcomes that may continue to improve over a longer period. Because these observations were derived from a small single-center cohort, they should be used to frame expectations rather than to predict the course of an individual patient.

The safety findings indicate that microdiscectomy has an acceptable risk profile in selected patients. The absence of complications, short hospital stay, and absence of mortality are consistent with those of previous pediatric microdiscectomy and minimally invasive surgical series (9, 10, 17, 25). Nevertheless, the occurrence of recurrence and reoperation indicates that early clinical success does not eliminate the need for long-term follow-up. Durham et al., Cahill et al., and Than et al. reported that recurrence and the need for revision surgery after pediatric LDH surgery were uncommon but possible (9, 10, 19).

### 4.6 Limitations

This study has several limitations. Its retrospective, single-center design and small sample size limit the precision and generalizability of the findings. With only 17 patients, the study was not adequately sized to support meaningful subgroup analyses according to age, disc level, herniation morphology, neurological deficit, activity profile, symptom duration, or other potentially relevant clinical variables. The procedures were performed by eight different neurosurgeons, which may have introduced within-cohort variability in operative technique and intraoperative decision-making. Conversely, the involvement of multiple surgeons may better reflect routine clinical practice than a single-surgeon series. The retrospective calculation of ODI scores and classification of modified MacNab outcomes from follow-up documentation may be susceptible to documentation and classification bias. Because patients treated non-operatively and a concurrent endoscopic surgery group were not included, comparative effectiveness could not be assessed. Although BMI status was classified using age- and sex-specific percentiles and reported descriptively, the small sample size and absence of a control group precluded assessment of BMI as a risk factor for lumbar disc herniation or as a predictor of postoperative outcomes. The radiological follow-up also had important limitations. Because CT was not performed systematically in all patients, the frequency of ring apophysis fracture may have been underestimated. Long-term segmental instability was not systematically evaluated because standardized postoperative dynamic radiographic surveillance was not part of the study protocol; therefore, the available clinical follow-up cannot exclude late radiographic instability. The responder analysis was *post hoc* and used thresholds derived from adult lumbar spine surgery populations because no MCID has been validated specifically for pediatric LDH. Moreover, the 30% criterion was developed for baseline-to-follow-up comparisons, and its application to change between 3 and 12 months was exploratory. Outcomes with a reference score of 0 were not evaluable for reduction and were excluded from the corresponding responder analyses. Accordingly, the reported recovery trajectories apply to this cohort and should be interpreted as exploratory observations rather than definitive estimates of postoperative recovery in the broader pediatric population. Larger prospective multicenter cohorts are needed to confirm these findings, assess subgroup-specific recovery patterns, and establish age-appropriate thresholds for clinically meaningful change. Despite these limitations, combining longitudinal repeated-measures analyses with patient-level absolute and relative responder assessments provides preliminary clinical context for the distinct components of recovery after pediatric and adolescent LDH surgery.

## 5 Conclusions

In conclusion, in this small single-center cohort, radicular leg pain, low back pain, disability, and global clinical outcome followed different postoperative trajectories after lumbar microdiscectomy. Radicular pain resolved early, whereas low back pain transiently increased immediately after surgery before subsequently declining. Functional recovery continued between 3 and 12 months, although the modified MacNab distribution did not show a statistically detectable change during the same interval. All evaluable patients met both adult-derived absolute and relative improvement criteria at 3 and 12 months, and the exploratory interval analysis suggested that proportional change was more sensitive than fixed absolute criteria in detecting continued late reduction in disability. These distinct temporal patterns may help clinicians counsel patients and parents regarding which aspects of recovery are likely to improve early and which may continue to progress over subsequent months. These findings apply to the present cohort and should be considered exploratory. Larger prospective multicenter studies are needed to confirm these observations, assess subgroup-specific recovery patterns, and establish pediatric-specific thresholds for clinically meaningful improvement.

## Data Availability

The data analyzed in this study are subject to the following licenses/restrictions: The dataset contains patient-level clinical and follow-up information and is therefore restricted due to privacy and ethical considerations. The data are not publicly available. Deidentified data may be made available by the corresponding author upon reasonable request, subject to institutional and ethical approval. Requests to access these datasets should be directed to erkanemrahoglu@gmail.com.
